# Diverticular peroral endoscopic myotomy: management of a complex epiphrenic diverticulum

**DOI:** 10.1055/a-2107-2287

**Published:** 2023-07-13

**Authors:** Abdelazeez Gaber, Ismail Fadloon, Hany Shehab

**Affiliations:** Integrated Clinical and Research Center for Intestinal Disorders (ICRID), Gastroenterology Division, Endemic Medicine Department, Cairo University, Cairo, Egypt

Epiphrenic diverticula are a rare entity presenting with dysphagia and vomiting in most cases. Conventional treatment usually involves complex thoracic surgeries with considerable morbidity, especially as the condition usually affects elderly patients. The overall mortality rate for surgical management of epiphrenic diverticula is about 5 %, with morbidity rate as high as 20 % 1 . A few case reports and case series have shown successful endoscopic management of epiphrenic diverticula by endoscopic septotomy (DPOEM) 2 3 . In this video we describe a case of a large complex epiphrenic diverticulum in a 78-year-old man presenting with long-standing dysphagia and vomiting ( Video 1 ).

**Video 1** Diverticular peroral endoscopic myotomy procedure to achieve septotomy in a large epiphrenic diverticulum.


A submucosal tunnel was created 5 cm above the diverticular septum. After reaching and exposing the septum, the tunnel was then extended laterally about 2 cm in the base of the diverticulum and medially down the esophageal aspect reaching 1 cm below the cardia ( Fig. 1 ). A full-thickness myotomy was then performed extending through the septum and down to involve the cardia (HybridKnife – Erbe VIO 3 generator with ERBEJET; mucosal incision and myotomy – Endocut Q 2:3:3; submucosal dissection – Precisect effect 4.5; Erbe Elektromedizin GmbH, Tübingen, Germany). Recovery was uneventful, and an oral diet was commenced after 24 hours. At the 3-month follow-up, symptoms of dysphagia and vomiting had completely resolved.

**Fig. 1:**
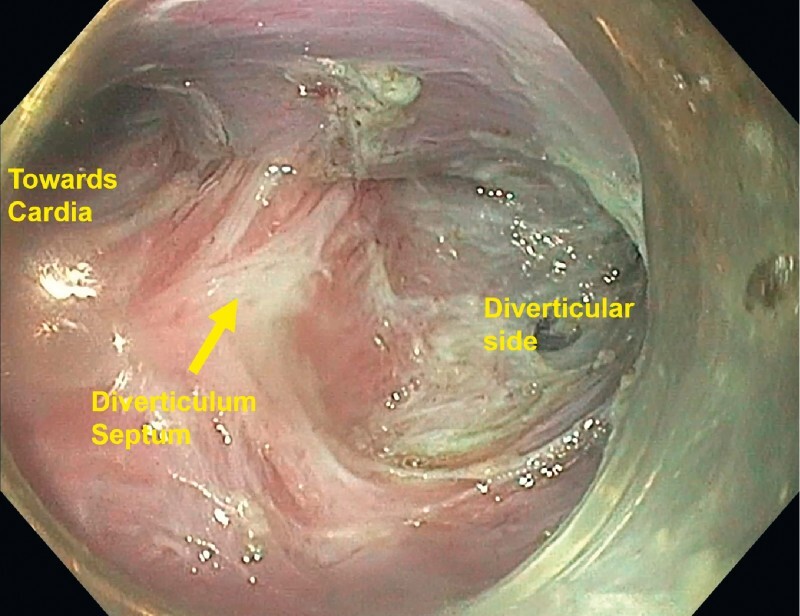
Exposure of the diverticular septum after submucosal tunneling.

Endoscopy_UCTN_Code_TTT_1AO_2AG

## Notes

Endoscopy E-Videos

https://eref.thieme.de/e-videos
*E-Videos* is an open access online section of the journal *Endoscopy* , reporting on interesting cases and new techniques in gastroenterological endoscopy. All papers include a high-quality video and are published with a Creative Commons CC-BY license. Endoscopy E-Videos qualify for HINARI discounts and waivers and eligibility is automatically checked during the submission process. We grant 100% waivers to articles whose corresponding authors are based in Group A countries and 50% waivers to those who are based in Group B countries as classified by Research4Life (see: https://www.research4life.org/access/eligibility/ ). This section has its own submission website at https://mc.manuscriptcentral.com/e-videos
